# A natural riboswitch scaffold with self-methylation activity

**DOI:** 10.1038/s41467-021-24193-7

**Published:** 2021-06-23

**Authors:** Laurin Flemmich, Sarah Heel, Sarah Moreno, Kathrin Breuker, Ronald Micura

**Affiliations:** University of Innsbruck, Institute of Organic Chemistry and Center for Molecular Biosciences (CMBI), Innrain 80-82, Innsbruck, 6020 Austria

**Keywords:** Biochemistry, RNA

## Abstract

Methylation is a prevalent post-transcriptional modification encountered in coding and non-coding RNA. For RNA methylation, cells use methyltransferases and small organic substances as methyl-group donors, such as *S*-adenosylmethionine (SAM). SAM and other nucleotide-derived cofactors are viewed as evolutionary leftovers from an RNA world, in which riboswitches have regulated, and ribozymes have catalyzed essential metabolic reactions. Here, we disclose the thus far unrecognized direct link between a present-day riboswitch and its inherent reactivity for site-specific methylation. The key is *O*^6^-methyl pre-queuosine (m^6^preQ_1_), a potentially prebiotic nucleobase which is recognized by the native aptamer of a preQ_1_ class I riboswitch. Upon binding, the transfer of the ligand’s methyl group to a specific cytidine occurs, installing 3-methylcytidine (m^3^C) in the RNA pocket under release of pre-queuosine (preQ_1_). Our finding suggests that nucleic acid-mediated methylation is an ancient mechanism that has offered an early path for RNA epigenetics prior to the evolution of protein methyltransferases. Furthermore, our findings may pave the way for the development of riboswitch-descending methylation tools based on rational design as a powerful alternative to in vitro selection approaches.

## Abstract (web summary)

In humans, protein methyltransferase is responsible for RNA methylation using S-adenosylmethionine as a methyl group donor. Here the authors report a self-methylation activity of a bacterial riboswitch.

## Introduction

In recent years, numerous discoveries of metabolite-sensing riboswitches hint at RNA World ribozymes that could have promoted chemical transformations^1–3^. One hypothesis suggests that present-day riboswitches for common enzyme cofactors may have evolved from ancient ribozymes that depended on these cofactors^4–7^. What has stood the test of time are RNA sensors of cofactors for the regulation of gene expression^8^ while the reactivity part has been taken over by more efficient protein-based modification machineries^9–12^. Riboswitches typically employ a highly conserved aptamer domain to sense the small molecule ligand with high specificity and selectivity, and an adjoining expression platform to regulate the expression of genes associated with ligand biosynthesis and transport^8,13,14^. Thereby, the partially overlapping RNA sequences of the two domains assist in converting the ligand-binding event into a change in gene expression by employing ligand-mediated RNA folding changes. It seems reasonable to assume that ligand-induced structural adaptions may also be exploited to trigger a chemical reaction. A single example exists for a known naturally occurring RNA, namely the *glms* riboswitch-ribozyme which binds glucosamine-6-phosphate whose protonated amino group then directly participates in reaction catalysis of RNA backbone cleavage^15^. Moreover, support for an advanced reactivity scope of cofactor-RNA systems has been provided by ribozyme engineering. The first methyltransferase ribozyme that catalyzes the site-specific installation of 1-methyladenosine in a substrate RNA, using a small-molecule cofactor has been created by in vitro evolution very recently^16^.

Numerous natural riboswitches are known to selectively sense nucleotide-derived metabolites connected to methyl-group transfer or one-carbon metabolism, comprising SAM, methylene tetrahydrofolate (THF) and adenosylcobalamin (vitamin B12)^17^. The aptamers of these riboswitches bind their dedicated ligands in the nano- to micromolar range. For all of them, different subclasses are found, meaning that the ligand is recognized by distinct RNA architectures with both specific binding pockets as well as overall folds^18^. For SAM-binding riboswitches, a total of six subclasses is known to date; they accommodate the ligand with its reactive methyl group in conformations that strictly avoid self-methylation^19,20^.

**Fig. 1:**
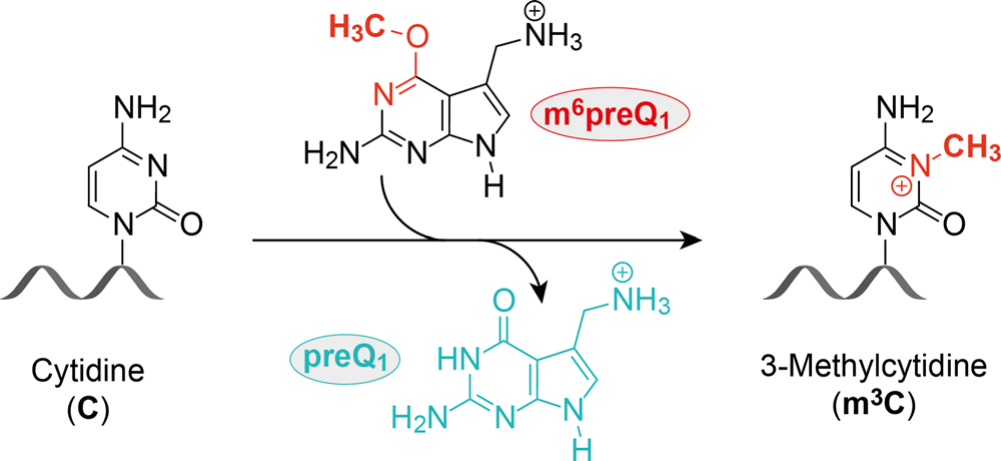
RNA methyltransferase activity. RNA sequence-specific installation of N3-methyl cytidine (m^3^C) using the small molecule m^6^preQ_1_ as cofactor has been discovered and is described in this study. The direct link between a present-day riboswitch scaffold and RNA-catalyzed methylation is reported.

Therefore, it has remained an open question whether riboswitches can catalyze site-specific methylation reactions to produce defined methylated RNA products^9,11^. Besides the above-mentioned ubiquitous enzyme cofactors, other small organic compounds could serve for RNA-catalyzed RNA methylation in modern riboswitches, as implicated by the recently in vitro selected RNA methyltransferase that utilizes *O*^6^-methyl guanine^16^. An obvious link between guanine riboswitches and the near-cognate ligand *O*^6^-methyl guanine was tested even prior to the in vitro selected methyltransferase, however, methylation was not observed^21^. Nevertheless, we speculated that other riboswitches sensing guanine-type ligands may possess the inherent reactivity for methyl group transfer that we were searching for. Here, we report on the identification of a riboswitch motif that uses *O*^6^-methyl pre-queuosine (m^6^preQ_1_) as a small-molecule methyl-group donor, catalyzing site-specific methylation of a cytosine at the N3-atom, resulting in RNA sequence-specific installation of 3-methylcytidine (m^3^C) (Fig. 1).

## Results

### Reactivity predictions for the preQ_1_ riboswitch and ligand requirements

**Fig. 2:**
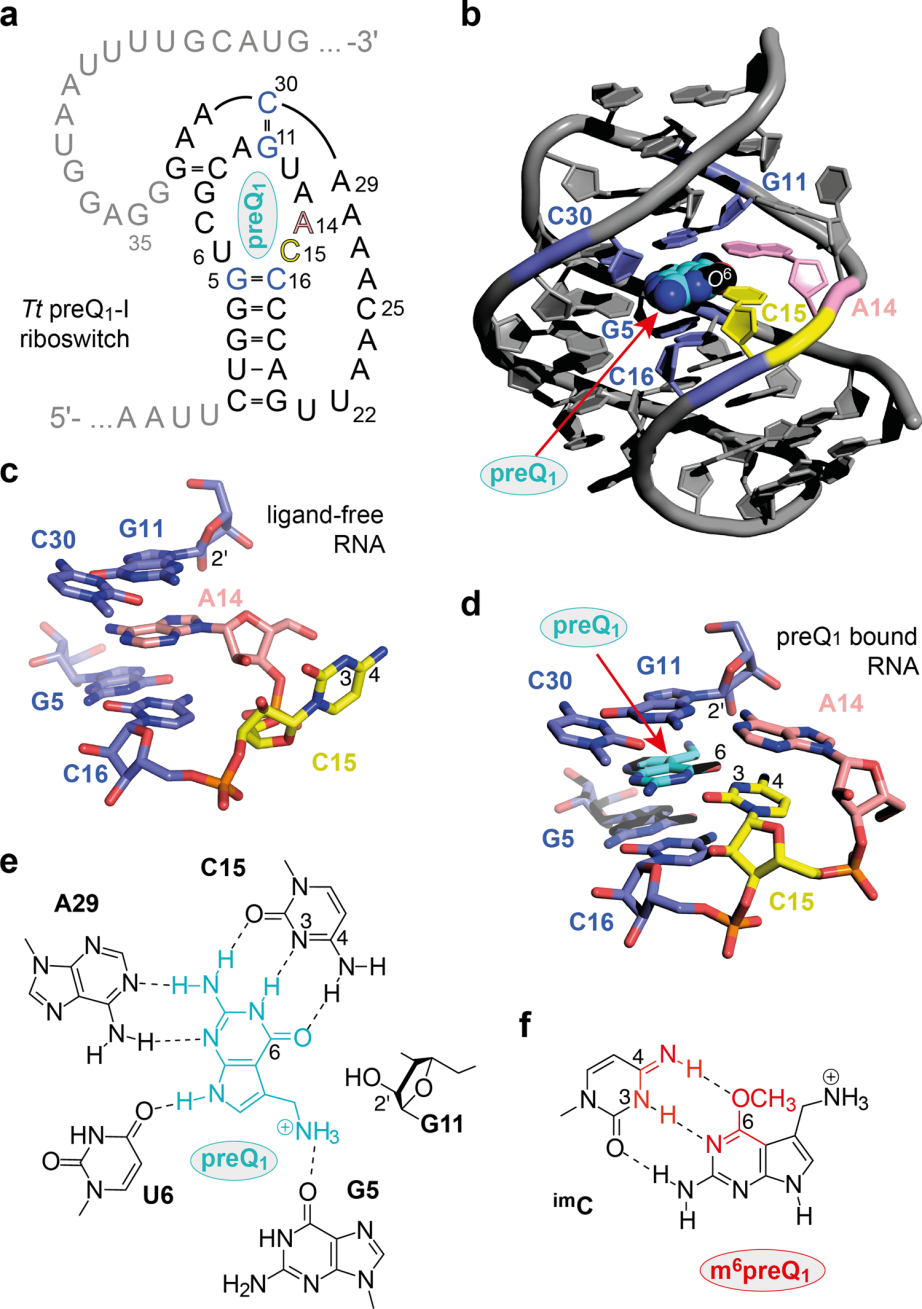
Analysis of preQ_1_-I riboswitch structures in the light of reactivity prediction. **a** Sequence and secondary structure of the *Tt* preQ_1_-I riboswitch (minimal aptamer motif in black). **b** Cartoon representation of the three-dimensional fold of the preQ_1_-bound aptamer (pdb code 3Q50; DOI: 10.2210/pdb3q50/pdb). **c** Stick representation of the ligand-free pocket revealing that A14 slides into the pocket and fills the space of preQ_1_ while C15 flips out and becomes exposed (pdb code 3Q51; DOI: 10.2210/pdb3q51/pdb). **d** Stick representation of the preQ_1_-bound riboswitch pocket with crucial atom numbers annotated in black. **e** Chemical structures illustrating the hydrogen bond network of preQ_1_ in the riboswitch pocket and the 2’-OH group of G11 that comes close the Hoogsteen face of preQ_1_. **f** Structure of a putative base pair between C15 (imino tautomer) and m^6^preQ_1_ in the preQ_1_-I riboswitch pocket.

We started our ambitious scheme to uncover RNA methylation activity with the analysis of ligand binding sites of various riboswitches and soon focused on preQ_1_ class I riboswitches (Fig. 2a) for which high-resolution crystal structures of both ligand-free and ligand-bound states were available (Fig. 2b, c)^22–24^. This choice was also guided by our hypothesis that the methylated version of the cognate ligand preQ_1_, namely m^6^preQ_1_, might be an excellent candidate for methyl group donation since m^6^preQ_1_ should retain the majority of interactions with the RNA as encountered in the genuine ligand-riboswitch system. Moreover, the principal reactivity of m^6^preQ_1_ is expected to be comparable to m^6^G that serves as cofactor in the recently in vitro selected RNA methyltransferase^16^.

Figure 2a shows the sequence and secondary structure of the 33 nt long aptamer of the *Thermoanaerobacter tengcongensis* (*Tt*) preQ_1_ riboswitch which adopts an H-type pseudoknot structure exhibiting low nanomolar affinity for preQ_1_ (Fig. 2b). The ligand is recognized by forming a Watson-Crick base pair with C15 (Fig. 2d,e) while the ligand’s N3 hydrogen acceptor and the C2-NH_2_ group are recognized in bidentate fashion via the Watson-Crick face of A29, complemented by a single H-bond between N9-H and the carbonyl oxygen of U6. Furthermore, a critical interaction responsible for the high affinity of preQ_1_ is attributed to the 7-aminomethyl group and the carbonyl oxygen of G5. The tight binding pocket is further defined by preQ_1_ stacking between the G5-C16 base pair and the nucleobase of G11 whose ribose 2′-OH is directed toward the preQ_1_ O6 atom and the 7-aminomethyl group (Fig. 2e). Importantly, the crystal structure of the ligand-free *Tt* RNA (Fig. 2c) points at the high flexibility and structural dynamics of parts of the binding pocket: C15 is now directed outwards while the nucleobase of its next neighbor (A14) slides into the stacked position filling the space of the former C15-preQ_1_ base pair.

Because of the above-highlighted characteristics in structure and structural dynamics of the preQ_1_ class I binding site we anticipated a high probability that the ligand congener m^6^preQ_1_ transfers its methyl group to the RNA: First of all, recognition of m^6^preQ_1_ by wild-type C15 should be easily possible if the tautomeric equilibrium is shifted towards the imino tautomer ^im^C15 (Fig. 2f). Second, the wild-type RNA pocket provides sufficient space to accommodate the methyl group (Fig. 2b,d). Third, there are three potential nucleophilic groups for methylation available in the vicinity of the methyl group if m^6^preQ_1_ becomes recognized by the pocket: these are the 2′-OH of G11, the C4-NH_2_ of C15, and the N3 of C15 (Fig. 2 d,e).

### Identification of methylated RNA product and reaction conditions

A methylation product of the preQ_1_ riboswitch would be difficult to distinguish from the unmodified RNA by gel shift or HPLC assays because its expected retention time will hardly differ. Therefore, we set out for an advanced mass spectrometric technology, Fourier-transform ion cyclotron resonance (FT-ICR) mass spectrometry (MS), which can determine the mass-to-charge ratio (m/z) of ions with very high precision by measuring the frequency of their cyclotron motion in a static magnetic field^25,26^. A major strength of such a set-up is that an RNA can be directly sequenced along with the identification of modifications and their localization (top-down RNA characterization) as we have demonstrated earlier^27^.

**Fig. 3:**
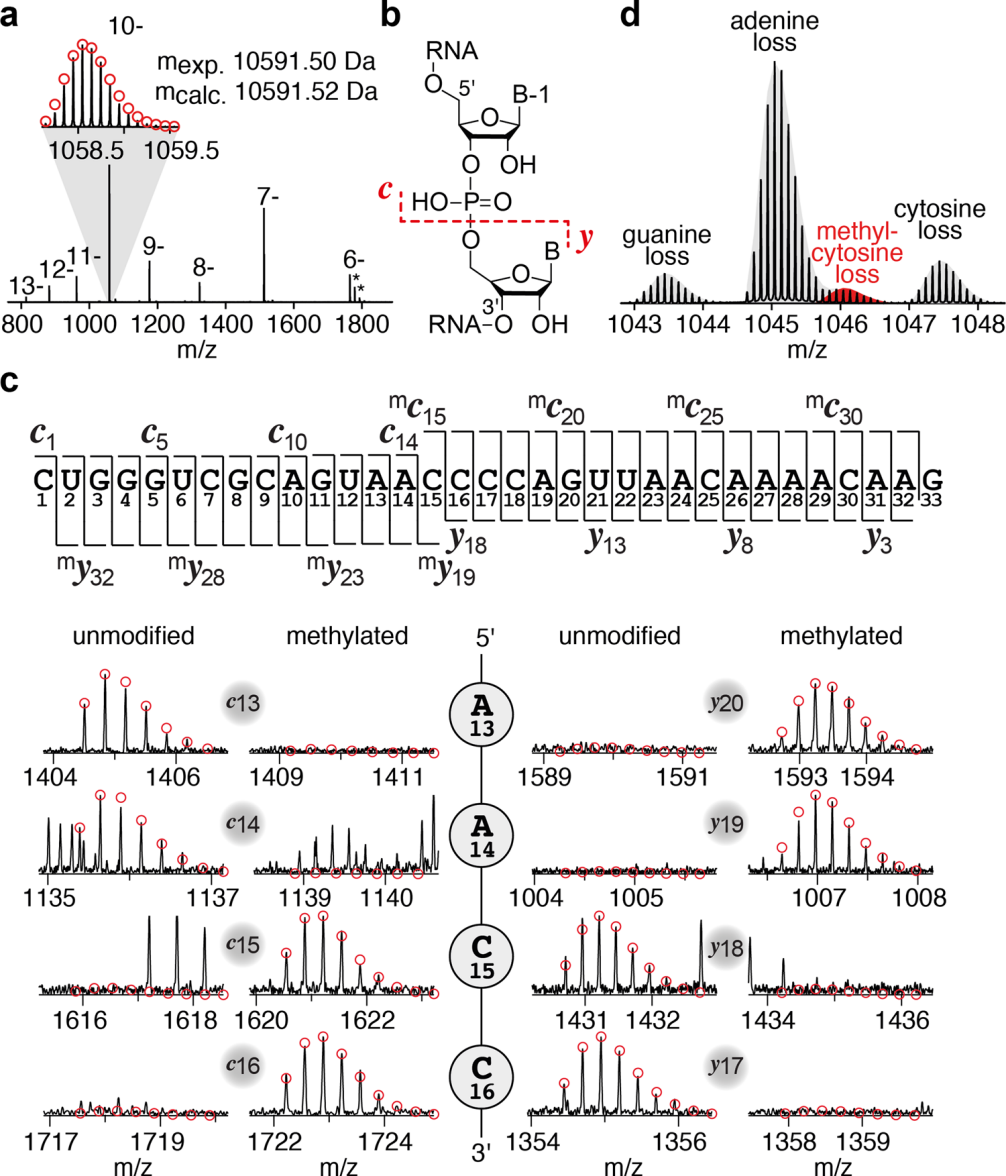
FT-ICR mass spectrometric characterization of the methylated RNA product. **a** ESI mass spectrum of the 33 nt m^3^C Tt preQ_1_-I RNA; the inset shows the isotopically resolved signal of the (M–10H)^10-^ ions; asterisks indicate piperidine RNA adducts. **b** Collisionally activated dissociation (CAD) of (M–nH)^n–^ ions of RNA in the collision cell produces *c* and *y* fragment ions from RNA backbone cleavage. **c** Fragment-ion map illustrating sequence coverage from CAD of the methylated *Tt* preQ_1_-I RNA. The numbering of c and y fragments starts from the 5’ and 3’ terminus, respectively (top). MS signals of unmodified and methylated c_13_, c_14_, c_15_, c_16_, and complementary y_20_, y_19_, y_18_, y_17_ fragments from CAD of (M−7H)^7−^ and (M–10H)^10−^ ions reveal the site of methylation (C15); the calculated isotopic profiles for unmodified and singly methylated RNA are indicated by red open circles. **d** Loss of methylated cytosine (red) in spectra from collisionally activated dissociation (CAD) of (M–10H)^10-^ ions of RNA is direct evidence for C nucleobase methylation; the 1:3 ratio of m^3^C/C nucleobase losses is consistent with destabilization of the glycosidic bond as a result of methylation at the N3 position.

We prepared the 33 nt long *Tt* preQ_1_ riboswitch by RNA solid-phase synthesis, synthesized the m^6^preQ_1_ ligand, and for initial trials, incubated both in a riboswitch-typical aqueous buffer system (2.5 µM RNA, 75 µM m^6^preQ_1_, 50 mM MOPS, 100 mM KCl, 2 mM MgCl_2_, pH 7.0). The reaction mixture was left overnight at room temperature, after which ultrafiltration with centrifugal concentrator devices was applied to remove the ligand and salts from the RNA for direct characterization by electrospray ionization (ESI) FT-ICR MS. Encouragingly, the high-resolution FT-ICR mass spectrum indeed showed the anticipated signal with a 14.0157 Da mass increase, consistent with a methyl group attached to the RNA (Fig. 3a). The power of FT-ICR mass spectrometry is further manifested in the fact that backbone cleavage by collisionally activated dissociation (CAD) produced a complete set of *c* and *y* fragments (Fig. 3b,c) that allowed for sequence determination (Fig. 3c). The fragment mass values unequivocally revealed the site of methylation at the C15 nucleoside. Furthermore, the loss of methyl-C from RNA ions (Fig. 3d) is direct evidence that the methyl group is located at the nucleobase. With respect to the precise position, the observed ratio for C to methyl-C of 3:1, which is higher than the statistical ratio of 8:1 according to the number of Cs in the sequence, suggests the N3 atom because methylation at the imino group generates a positive charge that weakens the glycosidic bond and in consequence facilitates base loss.

**Fig. 4:**
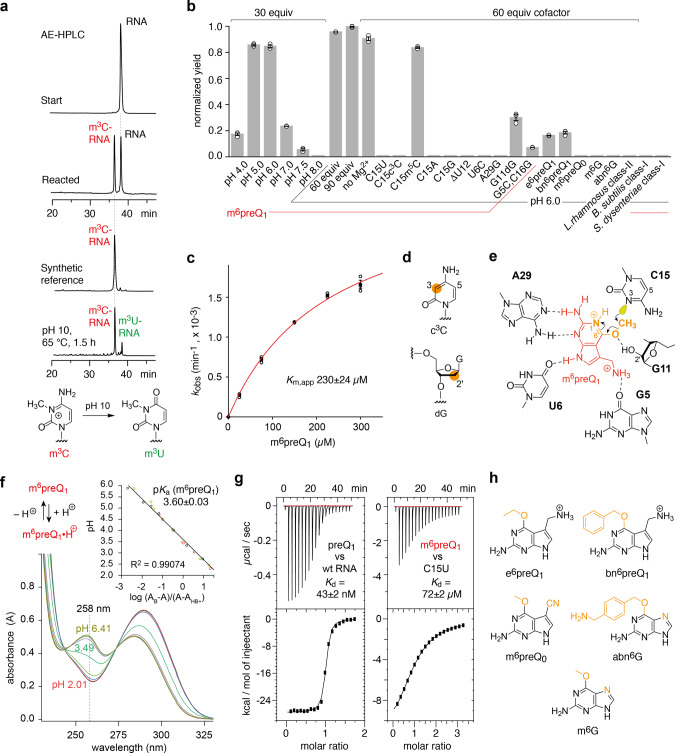
Reactivity scope of the *Tt* preQ_1_-I riboswitch scaffold. **a** Anion-exchange HPLC analysis of the preQ_1_-I RNA-catalyzed reaction with m^6^preQ_1_ at 37 °C; 2.5 μM RNA substrate, 225 μM m^6^preQ_1_, 2.0 mM MgCl_2_, pH 6.0. HPLC traces of unmodified RNA, the reacted mixture, and the corresponding m^3^C-modified synthetic RNA reference is shown for comparison (for reaction time course see Supplementary Fig. 1b). The m^3^C-modified RNA elutes earlier. Incubation of m^3^C preQ_1_-I RNA (isolated from a typical methylation reaction with m^6^preQ_1_) under harsh basic conditions produced m^3^U from m^3^C under concomitant RNA hydrolysis (for FT-ICR MS characterization of m^3^U-RNA see Supplementary Fig. 1e); chemical structures of m^3^C-to-m^3^U hydrolysis. **b** preQ_1_-I RNA reactivity analysis using diverse buffer conditions, nucleobase mutagenesis, atomic mutagenesis, and near-cognate cofactors; bars (gray) show mean ± s.e.m. (*n* = 3 independent experiments); HPLC traces are depicted in Supplementary Fig. 2, 3; source data are provided in the Source Data file. **c** The reaction rate is dependent on m^6^preQ_1_ concentration. The observed rate constants *k*_obs_ were determined based on HPLC trace analysis at five concentrations of m^6^preQ_1_, ranging from 25 to 300 μM. The red line represents a curve fit to *k*_obs_ = *k*_max_[m^6^preQ_1_]/(*K*_m,app_ + [m^6^preQ_1_]). Individual data points (open circles) (*n* = 3 independent experiments), mean ± s.e.m. (black circles); source data are provided in the Source Data file. **d** Chemical structures for c^3^C and dG used in atomic mutagenesis reactivity assay. **e** Proposed reaction mechanism of preQ_1_-I RNA–catalyzed methylation using m^6^preQ_1_ as cofactor; key features are the *syn*-conformation of the methyl iminoester moiety and N1 protonation of m^6^preQ_1_; furthermore, coordination of the 2’-OH G11 to *O*^6^ of m^6^preQ_1_ assists in cofactor alignment and contributes to improve leaving group quality. **f** UV-spectroscopic determination of the p*K*_a_ value of m^6^preQ_1_ (*n* = 3 independent experiments); source data are provided in the Source Data file. **g** Representative isotherm and ‘One set of sites’ binding­model fit for the preQ_1_ class I riboswitch titrated with preQ_1_ (left) and m^6^preQ_1_ (right); the indicated *K*_d_ values were determined at pH 6 (*n* = 3 independent experiments); source data are provided in the Source Data file; one experiment for preQ_1_ is depicted (left): *N* = 0.97, *K*_A_ = 2.37 × 10^7^ M^−1^, Δ*H* = −27.57 × 10^3^ cal mol^­1^, and Δ*S* = −58.7 cal (mol K)^­1^; one experiment for m^6^preQ_1_ is depicted (right): *N* = 0.99, *K*_A_ = 1.36 × 10^4^ M^−1^, Δ*H* = −12.85 × 10^3^ cal mol^­1^, and Δ*S* = −24.2 cal (mol K)^­1^. **h** Chemical structures of other potential cofactors tested.

To be able to rapidly analyze the methylation reaction, we optimized anion-exchange HPLC conditions, resulting in clear baseline separation of the methylation product from the unmodified RNA (Fig. 4a). We then tested different reaction conditions. Most importantly, when we decreased the pH from 7.0 to 6.0, the reaction yields increased to a significant extent, approaching 50% after 24 h which is the maximum yield possible, considering that the cognate preQ_1_ ligand (with low nanomolar affinity)^21,28^ released during the methylation reaction will be bound by the riboswitch/ribozyme, thereby blocking the binding of m^6^preQ_1_ (Fig. 4b and Supplementary Fig. 1a). We note that upon removal of the low molecular weight compounds by ultrafiltration, followed by adding a new batch of m^6^preQ_1_ the reaction yields were increased further (Supplementary Fig. 1a). Importantly, the reaction rate was dependent on m^6^preQ_1_ concentration, with an apparent Michaelis constant *K*_m_ of about 230 μM (Fig. 4c and Supplementary Fig. 1b). In this context, we mention that when a one-fold excess of m^6^preQ_1_ over RNA was applied only, methylation yields still amounted to 25% (Supplementary Fig. 1b).

To unequivocally confirm the site of methylation, we synthesized m^3^C phosphoramidite for RNA solid-phase synthesis and prepared the corresponding m^3^C15 containing RNA reference (Fig. 4a). This RNA had the same retention time as the product obtained by methylation using m^6^preQ_1_. We further point out that the synthetic m^4^C15 and m^5^C15 RNA references had retention times that were undistinguishable from the unmodified RNA in our HPLC assay (Supplementary Fig. 1c). Therefore, we separated the m^3^C RNA product from a methylation reaction mixture and analyzed the remaining RNA by FT-ICR mass spectrometry, but no further methylation products were detected (Supplementary Fig. 1d). Furthermore, we exposed the isolated m^3^C15 RNA to harsh basic conditions (pH 10) and observed the expected m^3^C-to-m^3^U conversion product (beside emerging fragments from RNA degradation) which additionally confirms that m^3^C15 RNA was formed selectively (Fig. 4a and Supplementary Fig. 1e).

### RNA sequence requirements and reaction mechanism

Next, we set out to investigate the *Tt* preQ_1_ class I riboswitch sequence requirements for methyl group transfer. We observed no methylation products when C15 was mutated either to 3-deazacytidine (c^3^C), U, A, or G (Fig. 4b,d and Supplementary Fig. 2). In contrast, when C15 was mutated to 5-methylcytidine (C15m^5^C), methylation occurred almost as efficiently as for wildtype RNA (Fig. 4b and Supplementary Fig. 2). Furthermore, when the bulged-out nucleotide U12 close to the binding site, was deleted (mutant ΔU12), no methylation occurred (Fig. 4b). U12, therefore, seems essential for retaining the structural dynamics within the U12-A13-A14-C15 segment. Moreover, when U6 and A29 which are crucial for recognition of the N9–N3 face of preQ_1_ and m^6^preQ_1_ were mutated individually to C and G, methylation was not observed anymore (Fig. 4b and Supplementary Fig. 2). Importantly, compensatory mutation of the conserved base pair G5-C16 into C5-G16 gave a sevenfold decrease in yields for the m^3^C-modified RNA (Fig. 4b), consistent with the crucial interaction of the *O*^6^-G5 atom and the protonated 7-aminomethyl group of preQ_1_ or m^6^preQ_1_. Strikingly, we found evidence for a potential role of the 2′-OH group of G11 in the methyl transfer reaction. When we deleted this functional group (dG11 mutant), reaction yields decreased to one-fourth compared to the wild-type RNA (Fig. 4b,d). This finding together with the above-described pH dependence of the reaction leads to the proposed reaction mechanism depicted in Fig. 4e. The favorable, slightly acidic pH value of 5–6 is consistent with saturating protonation of the 7-aminomethyl group and its recognition by G5. Importantly, it is also consistent with protonation of N1 of m^6^preQ_1_, thereby facilitating stabilization of the preQ_1_ leaving group (Fig. 4f). Our observation that methylation activity becomes impaired at pH values lower than 5 suggests protonation of N3 of C15, and hence the loss of nucleophilicity needed for an attack at m^6^preQ_1_. Furthermore, the model is compatible with stabilization of the *syn* conformation of the methyl iminoester by a hydrogen bond between m^6^preQ_1_ *O*^6^ and the 2′-OH of G11.

Finally, we set out to estimate the affinity of m^6^preQ_1_ to the riboswitch. Using isothermal titration calorimetry (ITC), we first determined the dissociation constant of the cognate preQ_1_ ligand to wildtype preQ_1_ RNA at pH 6 as reference (Fig. 4g). The *K*_d_^ITC^ value amounted to 43.0 ± 1.9 nM. We then determined the affinity of m^6^preQ_1_ to the unreactive C15U RNA mutant and obtained a *K*_d_^ITC^ value of 72.2 ± 2.1 µM at pH 6.0. This micromolar *K*_d_ likely reflects the portion of pairing strength that arises from the identical recognition of the C7-CH_2_NH_2_ moieties and the N9–N3–C2-NH_2_ faces of preQ_1_ and m^6^preQ_1_, respectively, by the pocket through A29 and U6. We note that A29G and U6C mutants were both inactive (Supplementary Fig. 2). Further, we believe that the actual affinity of m^6^preQ_1_ to wild-type (C15) RNA is higher than to the unreactive C15U mutant because the imino tautomer ^im^C15 as proposed in Fig. 2f could in principle provide a better match with respect to hydrogen donor-acceptor interactions compared to U15.

### Near-cognate cofactors and other preQ_1_ riboswitch scaffolds

Encouraged by our finding that a simple, methylated nucleobase can act as cofactor for riboswitch methylation, we set out to synthesize and test structural congeners of m^6^preQ_1_, namely *O*^6^-ethyl-preQ_1_ (e^6^preQ_1_), *O*^6^-benzyl-preQ_1_ (bn^6^preQ_1_), and *O*^6^-methyl-preQ_0_ (m^6^preQ_o_) (Fig. 4h and Supplementary Fig. 3). These derivatives, however, exhibited only very minor—or no—alkylation activities under optimized conditions (Fig. 4b and Supplementary Fig. 1f,g and Supplementary Fig. 3). Of further note, *O*^6^-methylguanine (m^6^G) and *O*^6^-(4-aminomethyl)benzylguanine (abn^6^G) did not result in detectable amounts of m^3^C15 RNA product (Fig. 4b). The observation that the potential methyl group donors m^6^G and m^6^preQ_0_ did not transfer their methyl groups can be rationalized by considering the significantly lower affinities of their non-methylated precursors, preQ_0_ (17-fold lower)^22^ and G (at least 25-fold lower)^29^ compared to the cognate ligand preQ_1_. Therefore, it is reasonable to assume that m^6^preQ_1_ is also significantly stronger bound compared to m^6^G and m^6^preQ_0_. It is the 7-aminomethyl substituent of preQ_1_ and m^6^preQ_1_ that has to be present together with its interaction partner, the G5-C16 base pair to reach high affinities^22^ and high methylation yields (see G5C-C16G mutant, Fig. 4b). We further note that e^6^preQ_1_ and bn^6^preQ_1_ which provide the 7-aminomethyl substituent exhibit lower yields likely because of steric hindrance of their larger *O*^6^ substituents with the binding pocket.

Next, we were wondering if representatives of the other known preQ_1_ riboswitch classes also possess self-methylation properties^29^. Both, preQ_1_ class II and III riboswitches have an identical architecture of their preQ_1_-bound pockets, however, this pocket architecture is different from class I^30,31^. Characteristically, class II and III apply a preQ_1_–C *trans* base pair instead of a *cis* Watson–Crick pair (class I), and significantly, the *O*^6^ atom of preQ_1_ is rather solvent-exposed with no obvious RNA functionalities for nucleophilic attack nearby. Nevertheless, we tested the well-studied preQ_1_ II riboswitch from *Lactobacillus rhamnosus* for methyl transfer of m^6^preQ_1_; as expected, methylation was not observed (Fig. 4b).

Noteworthily, class I preQ_1_ riboswitches are categorized into three subtypes, with the *Tt* riboswitch belonging to type 1^29^. We, therefore, complemented our analysis of RNA sequence requirements with a type 2 (*Bacillus subtilis*) and a type 3 preQ_1_ riboswitch (*Shigella dysenteriae*). Both revealed no methyltransferase activity (Fig. 4b).

## Discussion

Our study answers the long-standing question of whether contemporary riboswitches can act as cofactor-dependent ribozymes by a clear yes. Direct evidence became possible after careful analysis of three-dimensional riboswitch architectures in combination with rational ligand design and chemical intuition that finally led to the discovery of methyltransferase activity of the *Tt* preQ_1_ class I riboswitch. PreQ_1_ itself is a guanine-derived precursor of the nucleobase queuosine (Q) that is found in the wobble position of tRNAs containing the GUN anticodon^32^. The presence of Q in tRNAs improves their ability to read degenerate codons, while the absence of enzymes participating in Q biosynthesis or salvage results in deleterious phenotypes in many organisms^33,34^. Noteworthily, *O*^6^-methylated versions of the pre-queuosine heterocycle have been found in nature, in form of 2-amino-5-cyano-4-methoxypyrrolo[2,3-d]pyrimidine produced by *Streptomyces*^35^, and as the corresponding *N*^2^ -glyosylated natural products huimycin and dapiramicin^36^. Their occurrence hints at potential cellular availability of the near-cognate ligand m^6^preQ_1_ for preQ_1_ riboswitches. In this context it is also interesting that preQ_1_ and SAM aptamers have been found in a putative tandem riboswitch architecture which might hint at the regulatory utility of linking m^6^preQ_1_ biosynthesis to the concentrations of SAM and preQ_1_^29,35,36^. At this time, one can only speculate about the biological relevance of a riboswitch with potential self-methylation activity. Such a scenario implies that m^3^C methylation in the riboswitch pocket impairs recognition of the cognate ligand preQ_1_ and thereby impacts the gene regulatory function of the riboswitch. Thus far, the m^3^C modification itself is known for tRNAs in position 32 in diverse organisms^37,38^, ranging from yeast^39^, *S. Pombe*^40^, parasites such as *Trypanosoma brucei*^41^, to mice, rat, and human^42^. The modification has attracted additional interest when m^3^C has been identified as dynamic modification also in mRNA, and corresponding writer (METTL2, METTL6, METTL8) and eraser enzymes (ALKBH1, ALKBH3) have been characterized^43^. The emerging importance of the modification stimulated the development of novel RNA sequencing approaches, AlkAniline-Seq^44^ and HAC-seq^45^, respectively, to reveal m^3^C sites transcriptome-wide.

**Fig. 5:**
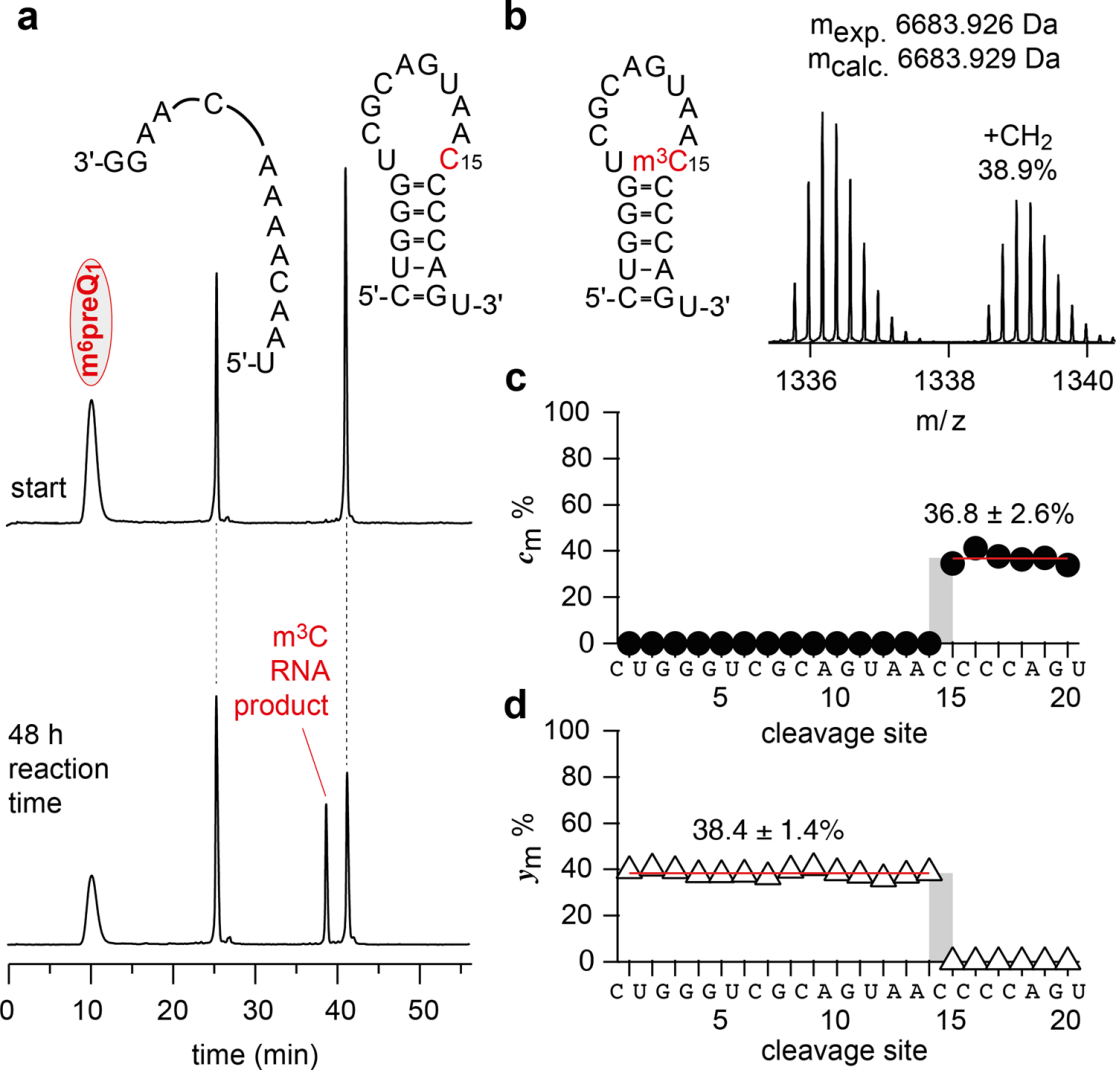
Methyltransferase activity using a split aptamer of preQ_1_-I RNA. **a** Sequences of the two *Tt* preQ_1_-I fragments used and anion-exchange HPLC traces of the reaction mixture at time points 0 and 48 h (25 μM each RNA strand, 1.5 mM m^6^preQ_1_, 2.0 mM MgCl_2_, 100 mM KCl, MES buffer pH 6.0, 37 °C); as expected, the m^3^C-modified RNA product elutes earlier. **b** Secondary structure of the m^3^C-modified preQ_1_-stem-loop RNA and CAD-MS of co-isolated (M − 5H)^5-^ ions of unreacted 21 nt RNA (61.1%, *m*_exp._ 6683.926 Da, *m*_calc._ 6683.929 Da) and methylated RNA (38.9%, *m*_exp._ 6697.942, *m*_calc._ 6697.945 Da) produced at 65 eV laboratory frame energy *c* and *y* fragments with and without methylation. **c** Thereby, the fraction of methylated *c* fragments (*c*_m_) increased from 0% to 36.8 ± 2.6% at site 15 (mean of values for sites 15 to 20 ± standard deviation). **d** The fraction of methylated *y* fragments (*y*_m_) decreased from 38.4 ± 1.4% to 0% at site 15 (mean of values for sites 1 to 14 ± standard deviation), which both are consistent with C15 as methylation site and with the yields independently obtained by HPLC analysis. Source data are provided in the Source Data file.

We furthermore anticipate that the discovered methyltransferase activity of the preQ_1_ class I riboswitch can serve as starting point for engineering in vitro and in vivo methylation tools. Applications for m^3^C installation are conceivable not only in cis, but also in trans. First experimental attempts towards this aim by splitting the aptamer into two halves have been successful and led to remarkable 38% of methylation for the preQ_1_ stem-loop by simply adding the 3′-terminal preQ_1_ RNA fragment in the presence of cofactor (Fig. 5). Beyond methyltransferase activity and the particular m^3^C modification investigated here, our work may stimulate the search for other ribozyme-catalyzed reactions that are lying dormant in present-day riboswitches. Given the large number and great diversity of riboswitches that have been identified to date, a broad repertoire of chemical reactivities should be directly retrievable without the need for nucleic acids evolution that builds on constructed RNA libraries of naturally occurring riboswitch scaffolds^46^ or on de novo in vitro RNA selection approaches^47^.

Finally, we stress the importance of our findings for the RNA world hypothesis which postulates that possible early life on Earth relied on catalytic RNA properties essential for evolving sophisticated RNA-based life^5–10,48^. The idea is strongly supported by the original discovery of bacterial riboswitches that regulate—without the aid of proteins—the expression of genes that are involved in the biosynthesis of metabolites^6^. Some of these riboswitches may have evolved from ancient cofactor-dependent ribozymes preserving their regulatory capacity while the reactivity part has been taken over by more efficient protein enzyme machineries. Our findings may also hint at a prebiotic version of RNA epigenetics with methylated nucleobases taking the role as cofactors for ancient methyltransferase ribozymes^49–52^.

## Methods

### RNA synthesis

RNA oligonucleotides were prepared by solid-phase synthesis using phosphoramidite chemistry (2′-*O*-TOM-protected) on controlled-pore glass solid supports^53^. RNA sequences are given in Supplementary Table 1. Modified phosphoramidites for atomic mutagenesis and reference oligonucleotides were purchased (ChemGenes, Glen Research, Berry Asscociates) or prepared in-house, following published procedures^54,55^. RNA oligonucleotides were deprotected with ammonia and butylamine instead of ammonia and methylamine to avoid any possible methylated RNA from deprotection, followed by 1 M tetrabutylammonium fluoride in THF, desalted (Sephadex G25), and purified by denaturing anion exchange chromatography (Dionex DNAPac PA100, 9 × 250 mm, at 80 °C; solvent A was 25 mM Tris-HCl (pH 8.0) and 20 mM NaClO_4_ in 20% aqueous acetonitrile; solvent B was 25 mM Tris-HCl (pH 8.0) and 0.6 M NaClO_4_ in 20% aqueous acetonitrile; the gradient was: linear, 25–40% (25–45 for longer sequences) with slope of 5 % solvent B per column volume). Customized deprotection conditions were used for m^3^C RNA (3:1 (v/v) 28–30% aqueous NH_3_ and EtOH, at 40 °C for 5 h), m^4^C RNA (O^4^-chlorophenyl U convertible nucleoside approach, 7 M NH_3_ in MeOH at 42 °C for 18 h). The quality of RNAs (purity and identity) was analyzed by anion-exchange HPLC (Dionex DNAPac PA100, 2 × 250 mm, eluents as above, the gradient was: linear, 22–35% solvent B, with slope of 0.87% solvent B per column volume), and HR–ESI–MS (Thermo Fisher Orbitrap, negative-mode) or FT ICR MS (see below). Measured and calculated masses are listed in Supplementary Table 1.

### Characterization of m^6^preQ_1_, e^6^preQ_1_, and bn^6^preQ_1_

Analytical data (NMR spectra) of m^6^preQ_1_, e^6^preQ_1_, and bn^6^preQ_1_ are shown in Supplementary Figs. 4–11. m^6^G and abn^6^G were purchased from commercial sources (Carbosynth).

### Reaction of preQ_1_ RNA with m^6^preQ_1_ or analogs

A typical methylation reaction was carried out in a volume of 200 μl containing 0.5 nmol RNA (2.5 µM) and 75 μM ligand (30 equiv) in buffer solution (2 mM MgCl_2_, 100 mM KCl, 50 mM 2-(*N*-morpholino) ethanesulfonic acid (MES), pH 6.0). After heating the mixture to 90 °C for 2 min, it was incubated for 48 h at 37 °C. After desalting using a Sep-Pak C18 cartridge, the methylated RNA product was directly analyzed in the mixture or isolated by AE HPLC, desalted again (Sep-Pak C18), and subjected to FT ICR-ESI-MS (which included a further desalting step using centrifugal concentrators; see below). For experiments at pH 4 and 5, sodium acetate buffer was used instead of MES; for pH 7, 7.5, and 8, MOPS was used. Several of the experiments were additionally analyzed using 60 and 120 equiv of the cofactor.

The hydrolysis of m^3^C to m^3^U RNA was examined in a volume of 280 μl with 7 nmol RNA (25 µM) in 25 mM Na_2_CO_3_ buffer (pH 10) with 1 mM EDTA at 65 °C for 1.5 h. After desalting (Sep-Pak C18) the m^3^U RNA was isolated by AE HPLC and analyzed by FT ICR–ESI–MS (see below).

### FT-ICR mass spectrometric analysis of RNA methylation/alkylation products

Methanol was HPLC grade (Acros), ammonium acetate (≥99.0%, Na ≤5 mg/kg, *K* ≤ 5 mg/kg), piperidine (≥99.5%), and imidazole (≥99.5%, Na ≤ 50 mg/kg, *K* ≤ 50 mg/kg) were from Sigma-Aldrich, and H_2_O was purified to 18 MΩ·cm at room temperature using a Milli-Q system (Millipore). Experiments were performed on a 7T Fourier transform ion cyclotron resonance (FT-ICR) mass spectrometer (Bruker APEX ultra) equipped with an ESI source for (M − *n*H)^n−^ ion generation and a collision cell through which a flow of Ar gas was maintained for CAD. The mass resolving power of this instrument is routinely (broadband detection, 2 M data points for a ∼2 s transient) ∼220,000, ∼120,000, and ∼80 000 at *m*/*z* 500, 1000, and 1500, respectively, and the mass accuracy is ∼1 ppm with internal calibration and ∼20 ppm with external calibration (Supplementary Table 1). RNA was electrosprayed (flow rate 1.5 µl/min) from 1–2 µM solutions in 1:1 or 9:1 H_2_O/CH_3_OH vol/vol with piperidine (2–10 mM) and imidazole (0–10 mM) as additives. Prior to dissociation by CAD, the (M − *n*H)^*n−*^ ions under study were isolated in a linear quadrupole; for a more detailed description of the experimental setup for CAD see^56^. For statistical reasons, between 25 and 500 scans were added for each spectrum (20–50 for ESI, 100–500 for CAD), and data reduction utilized the SNAP2 algorithm (Bruker). For desalting, 400 µl of an ammonium salt solution (100 mM ammonium acetate in H_2_O) was added to 100 µl RNA solution (about 1 nmol in H_2_O) and concentrated to 100 µl using Vivaspin 500 centrifugal concentrators (Sartorius, MWCO 3000). The process was repeated five to seven times, followed by six to seven cycles of concentration and dilution with H_2_O. RNA concentration was determined by UV absorption at 260 nm using a NanoPhotometer (Implen).

### Kinetic assays of RNA-catalyzed methylation reactions

The methylation reactions were carried out as described above (on a 1.5 nmol scale; 600 µl reaction volume) and 50-μl aliquots were taken at desired time points and quenched immediately by adding 50 μl of stop solution (12 M urea). The samples were analyzed by AE HPLC and UV-detection. The peak areas were quantified. The yield versus time data were fit to (fraction unreacted) = *Y*(1 − e^–*k*t^), in which *k* = *k*_obs_ and *Y* = final yield, using OriginPro (2020). All kinetic assays were carried out as three independent replicates.

### Isothermal titration calorimetry (ITC)

ITC measurements were performed on a MicroCal iTC_200_ instrument at 20 °C in 50 mM MES, 100 mM KCl and 2 mM MgCl_2_ (pH 6.0). For preQ_1_, the concentration of *Tte* preQ_1_ class I RNA in the measuring cell was typically 0.012 mM; preQ_1_ ligand was in the syringe at a concentration that was 10-fold higher than the RNA. The c-value was typically around 300. For m^6^preQ_1_, the concentration of the *Tte* preQ_1_ class I RNA in the measuring cell was typically 0.19 mM; m^6^preQ_1_ cofactor was in the syringe at a concentration that was 15-fold higher than the RNA. The *c*-value was typically around 3. Both *K*_d_ determinations were carried out as three independent replicates.

### Reporting summary

Further information on research design is available in the Nature Research Reporting Summary linked to this article.

## Supplementary information

Supplementary Information

Reporting Summary

## Data Availability

All relevant data are available from the corresponding author on reasonable request. Source data for calculation of errors in Figs. 4b, 4c, 4f, 4g, 5a, 5c, and 5d and Supplementary Fig. 1b, are provided as a Source Data file.

## Code availability

Custom software that would need code availability was not used for this study. Software used for data collection: Bruker Daltronics Compass Apex Control 3.0.0, Bruker Topspin 3.6, UNICORN 5.0, Chromeleon 6.8. Software used for data analysis: Microsoft Excel 16.16.22, OriginPro 2020.
