# Supplementary material for: A natural riboswitch scaffold with self-methylation activity
Source: Nat Commun. 2021 Jun 23;12:3877. doi: 10.1038/s41467-021-24193-7 (PMC8222354; doi:10.1038/s41467-021-24193-7)
Supplement: Supplementary file 1 — Supplementary Information [file 41467_2021_24193_MOESM1_ESM.pdf]

SUPPLEMENTARY INFORMATION

to

A natural riboswitch scaffold with self-methylation activity

Laurin Flemmich[1], Sarah Heel[1], Sarah Moreno[1], Kathrin Breuker[1], Ronald Micura[1]*

[1] University of Innsbruck, Institute of Organic Chemistry and Center for Molecular Biosciences (CMBI), Innrain 80-82, 6020 Innsbruck, Austria.

* e-mail: ronald.micura@uibk.ac.at

Contents

Supplementary Figure 1	2
Supplementary Figure 2	4
Supplementary Figure 3	6
Supplementary Figure 4	7
Supplementary Figure 5	8
Supplementary Figure 6	9
Supplementary Figure 7	10
Supplementary Figure 8	11
Supplementary Figure 9	12
Supplementary Table 1	13

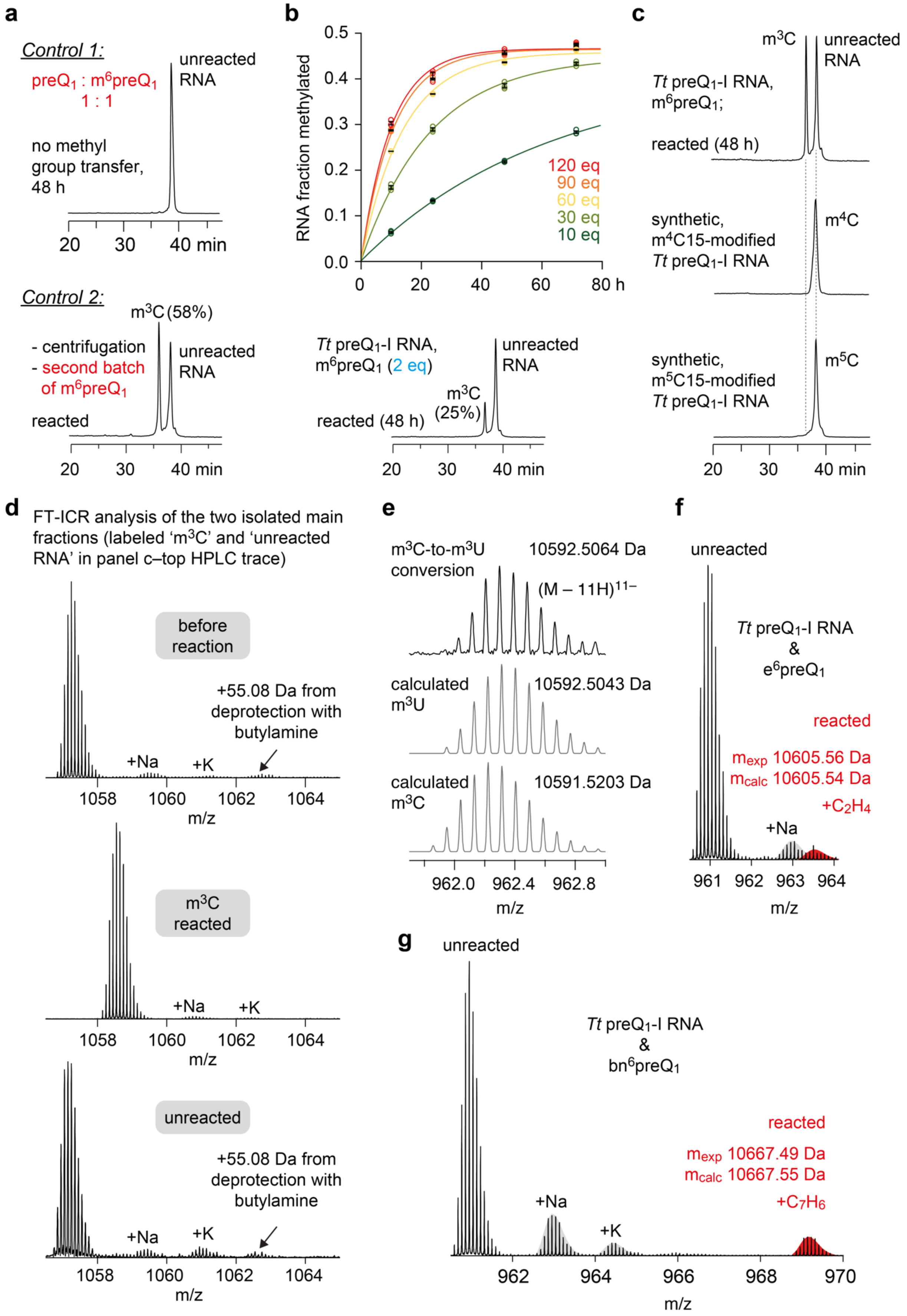

Supplementary Fig. 1 | Analysis of methyltransferase activity of *Tt* preQ$_1$-I riboswitch RNA. a, Inhibition of the methylation reaction is observed when m^{6}preQ$_1$ and preQ$_1$ are provided in 1:1 ratio (60 equiv each; under otherwise optimal reaction conditions) consistent with high affinity binding of

$preQ_1$ over m^6preQ_1 (control 1); methylation yields can be increased upon removal of the low molecular weight compounds by centrifugation (Vivaspin), followed by addition of a new batch of m^6preQ_1 the reaction (control 2). **b**, Kinetics of RNA-catalyzed self-alkylation of using m^6preQ_1 cofactor (2.5 µM RNA; 25, 75, 150, 225, or 300 µM m^6preQ_1; 2.0 mM $MgCl_2$; pH 6.0, 37 °C). Fraction modified is shown as mean ± s.d. (n = 3 independent experiments for each ligand concentration), and fit to a mono-exponential model ($Y = Y_{max}(1 - e^{-kt})$) (top); HPLC analysis of the methylation reaction with only one-fold excess of m^6preQ_1 over RNA (25 µM RNA; 50 µM m^6preQ_1; 2.0 mM $MgCl_2$; pH 6.0, 37 °C) (bottom). **c**, Comparison of retention times of a methylation reaction mixture (top) with synthetic m^4C15 (middle) and m^5C15 (bottom) modified RNA. Since putative m^4C RNA products would elute simultaneously as unmodified RNA, the reaction mixture was separated and the slower migrating fraction analyzed by FT-ICR mass spectrometry: no methylated RNA was detectable and m^4C excluded as the methylation product. **d**, FT-ICR mass spectrometric analysis of the separated methylation product and unreacted RNA from a typical reaction mixture to evaluate if other potential methylation products (e.g. m^4C RNA) were co-eluting with the unreacted RNA; this was not the case. **e**, FT-ICR mass spectrometric analysis of the hydrolysis products of isolated m^3C RNA exposed to strong basic conditions (pH 10). The 1-Dalton difference in molecular weight is consistent with m^3U RNA formation. **f**, FT-ICR mass spectrometric analysis of the C15 N3-ethylated product from the reaction mixture of e^6preQ_1 and *Tt* $preQ_1$-I RNA. **g**, FT-ICR mass spectrometric analysis of the C15 N3-benzylated product from the reaction mixture of bn^6preQ_1 and *Tt* $preQ_1$-I RNA.

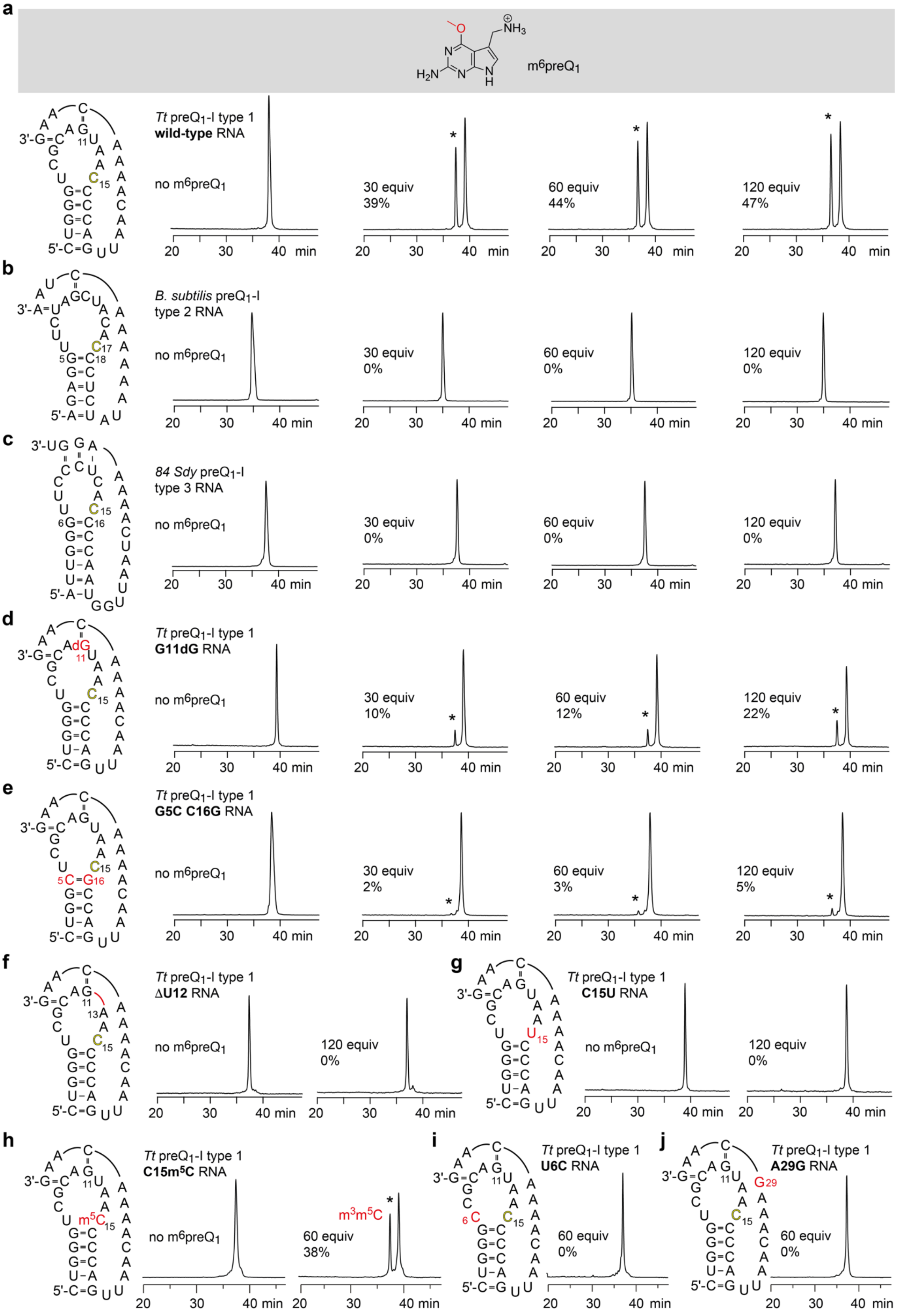

Supplementary Fig. 2. Anion-exchange HPLC analysis of the preQ$_1$-I RNA-catalyzed reaction with m^{6}preQ$_1$ at 37 °C; 2.5 µM RNA, 2.0 mM MgCl$_2$, pH 6.0; concentration of m^{6}preQ$_1$ as indicated, the

reaction yield plateaus after 24 h reaction time (see Supplementary Fig. 1a), the time point at 48 h is depicted. Methylated products are indicated by asterisks. RNAs investigated were **a**, *Thermoanaerobacter tengcongensis* preQ_1-I type 1 wildtype. **b**, *Bacillus subtilis* preQ_1-I type 2 wildtype. **c**, *Shigella dysenteriae* preQ_1-I type 3 wildtype. **d**, *Tt* preQ_1-I type 1 G11dG mutant. **e**, *Tt* preQ_1-I type 1 G5C G16C mutant. **f**, *Tt* preQ_1-I type 1 ΔU12 mutant. **g**, *Tt* preQ_1-I type 1 C15U mutant. **h**, *Tt* preQ_1-I type 1 C15m^{5}C mutant. **i**, *Tt* preQ_1-I type 1 U6C mutant. **j**, *Tt* preQ_1-I type 1 A29G mutant.

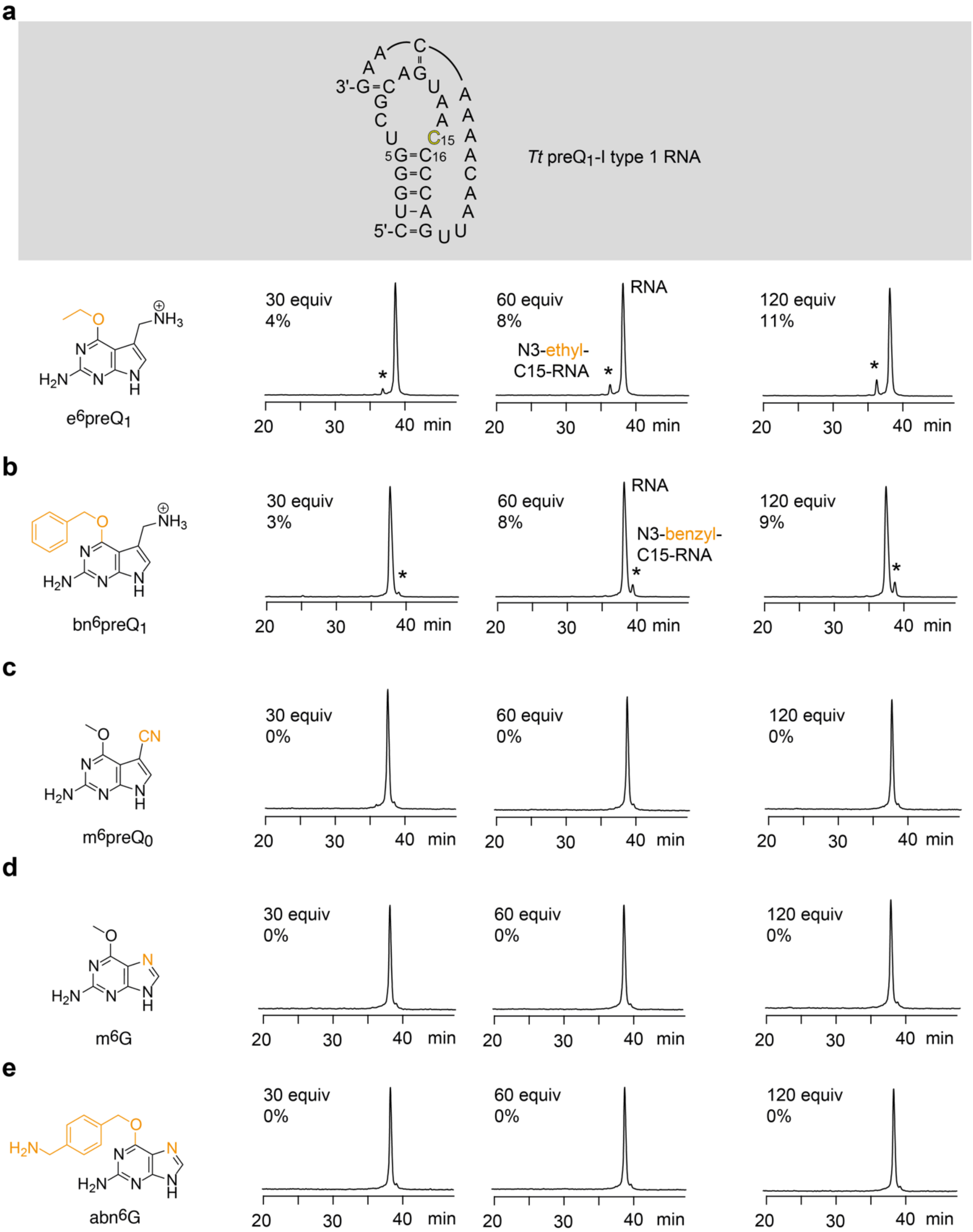

Supplementary Fig. 3. Anion-exchange HPLC analysis of the *Tt* $preQ_1$-I type 1 RNA-catalyzed reaction with diverse ligands at 37 °C; 2.5 µM RNA, 2.0 mM $MgCl_2$, pH 6.0; concentration of ligand as indicated; the reaction yield plateaus after 24 h reaction time (see Supplementary Fig 1a), the time point at 48h is depicted. Alkylated products are indicated by asterisks. Ligands investigated were **a**, e^6preQ_1 (for FT-ICR mass spectrum see Supplementary Fig. 1f). **b**, bn^6preQ_1 (for FT-ICR mass spectrum see Supplementary Fig. 1g). **c**, m^6preQ_0. **d**, m^6G. **e**, abn^6G.

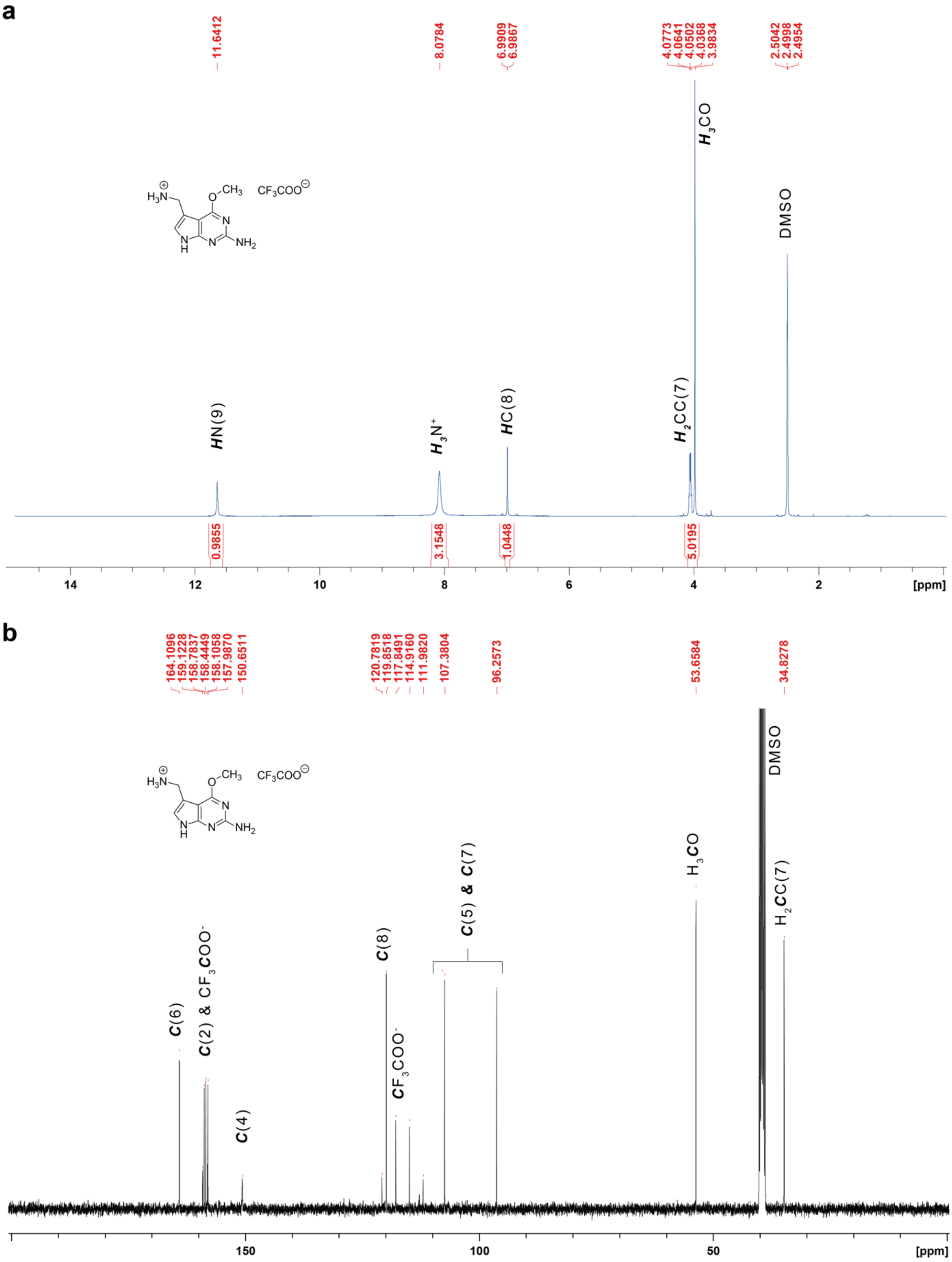

Supplementary Fig. 4. NMR spectroscopic analysis of m^{6}preQ$_1$ ligand. **a**, ^{1}H NMR spectrum. **b**, ^{13}C NMR spectrum.

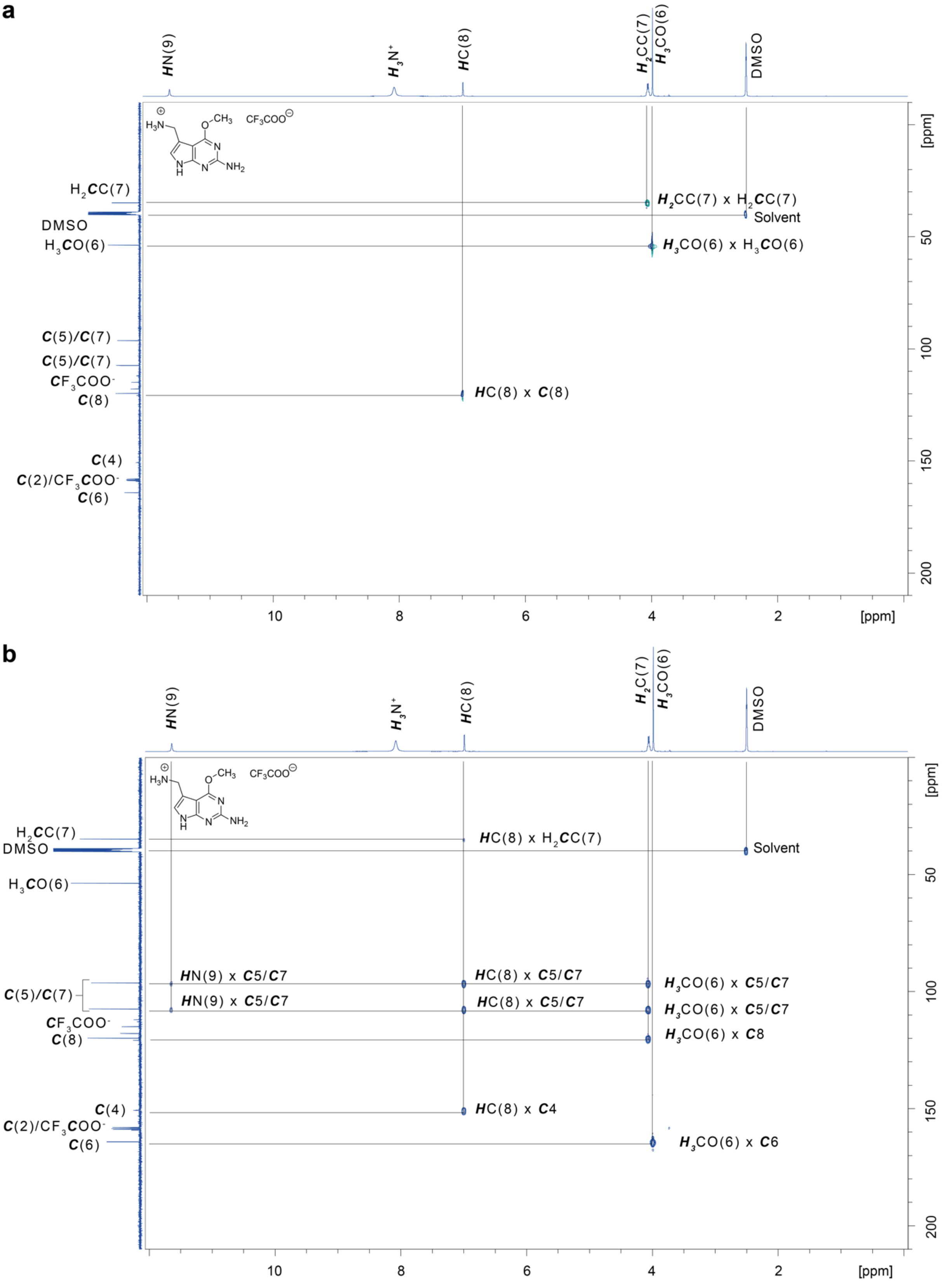

Supplementary. 5. NMR spectroscopic analysis of m^{6}preQ$_1$ ligand. **a**, ^{1}H,^{13}C HSQC NMR spectrum. **b**, ^{1}H,^{13}C HMBC NMR spectrum.

a

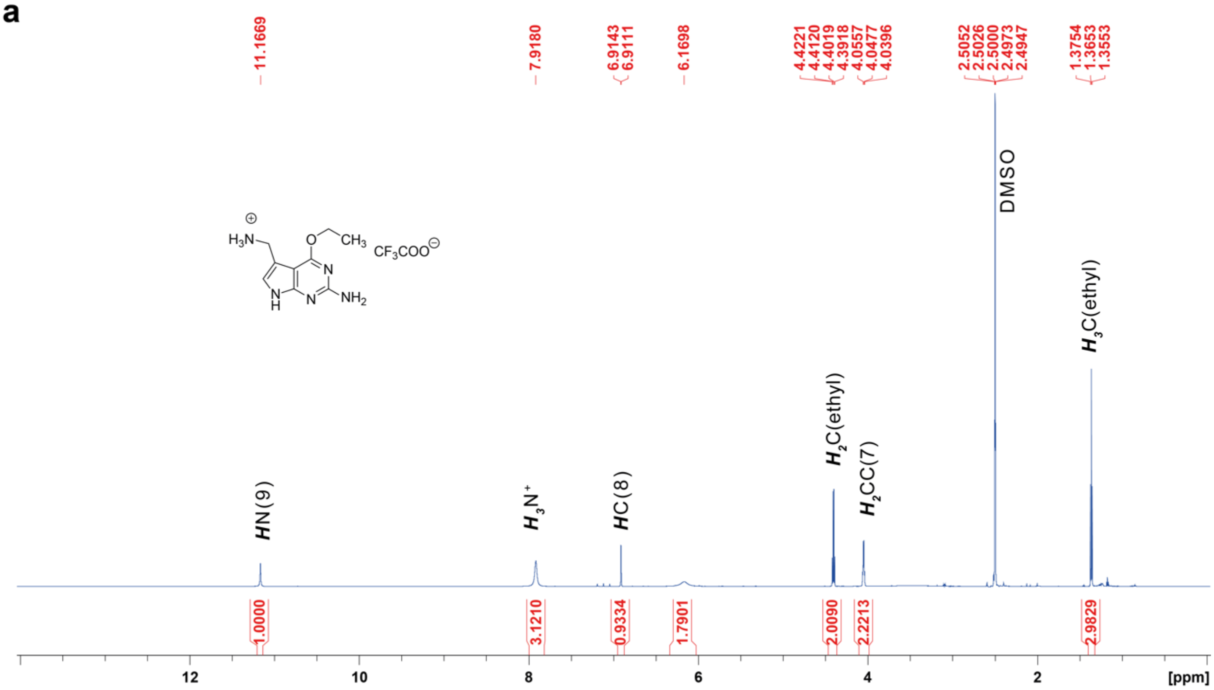

b

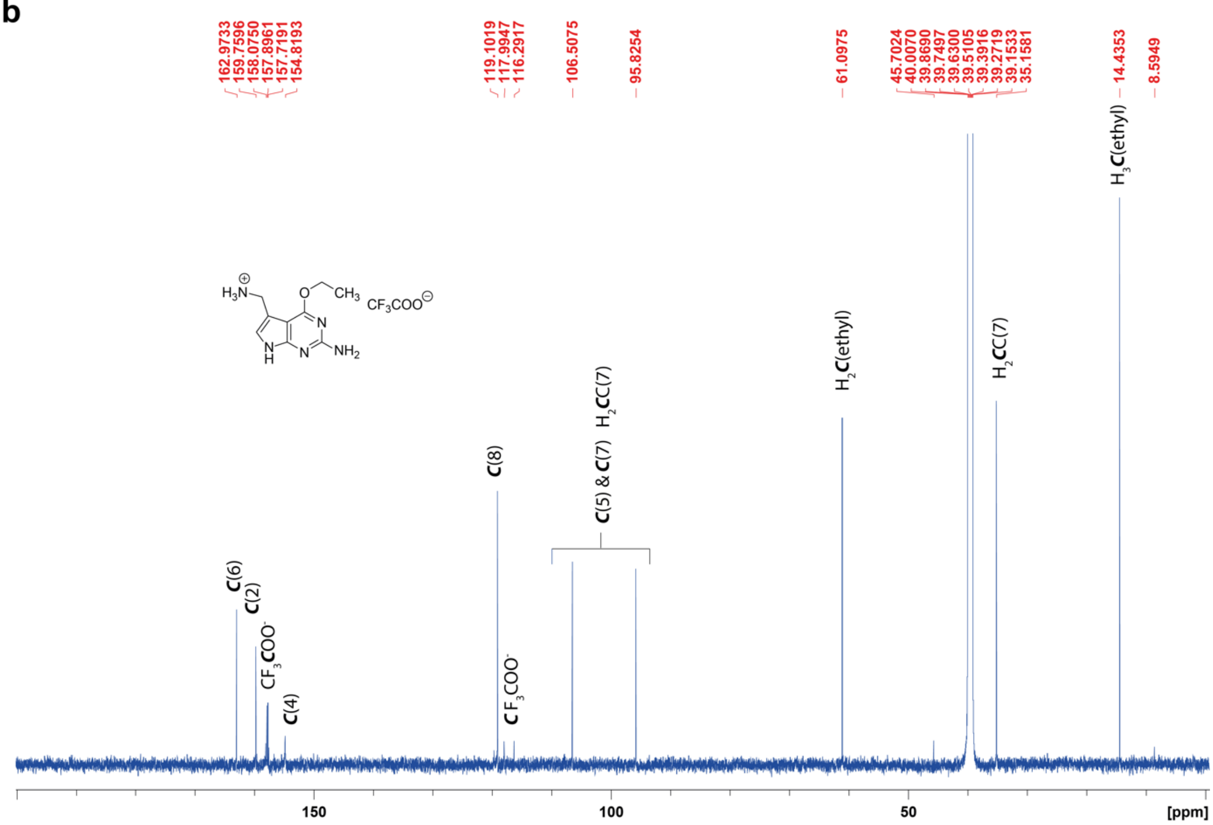

Supplementary Fig. 6. NMR spectroscopic analysis of e^6preQ_1 ligand. **a**, ^1H NMR spectrum. **b**, ^{13}C NMR spectrum.

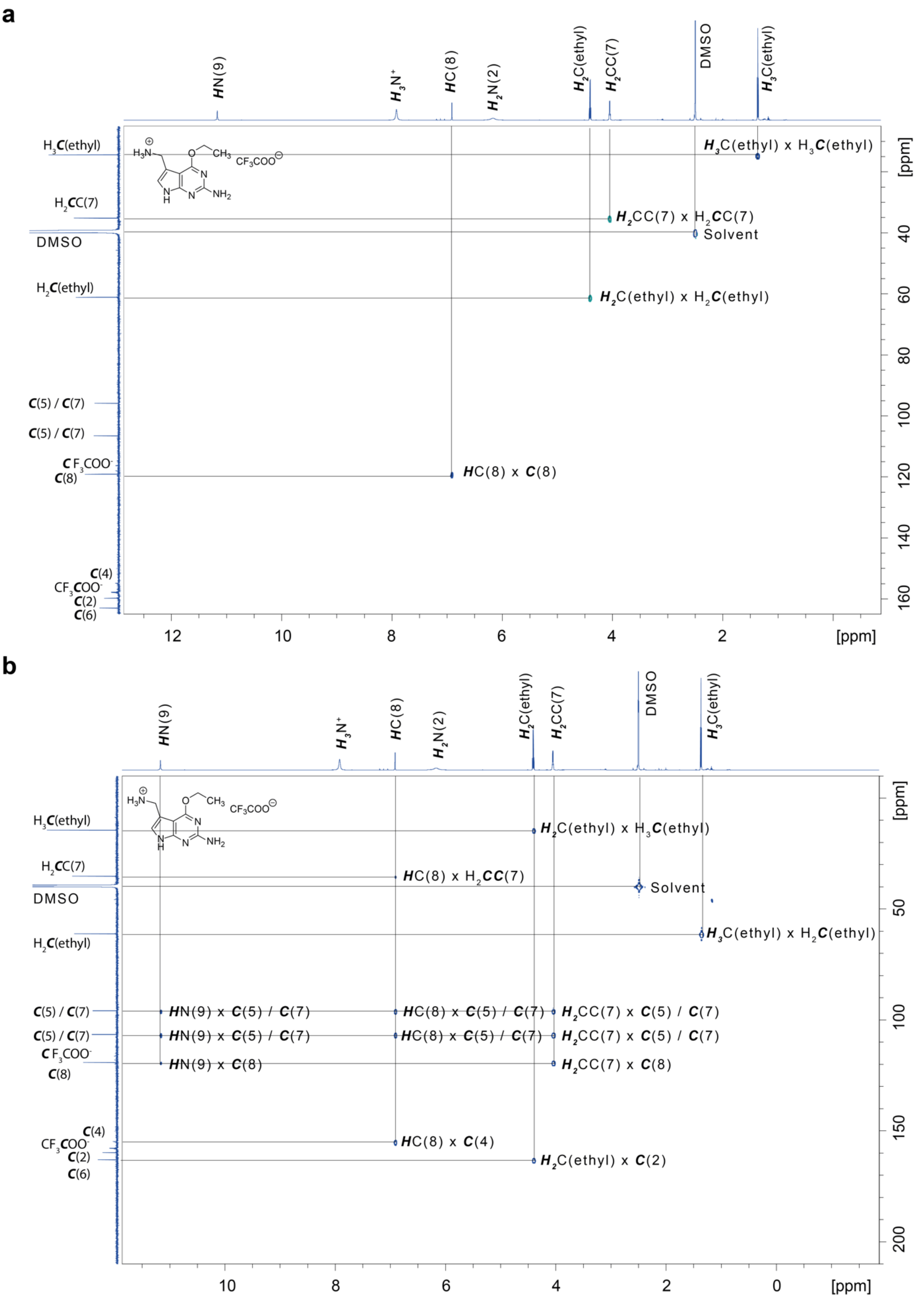

Supplementary Fig. 7. NMR spectroscopic analysis of e[6]preQ1 ligand. **a**, $^{1}H,^{13}C$ HSQC NMR spectrum. **b**, $^{1}H,^{13}C$ HMBC NMR spectrum.

a

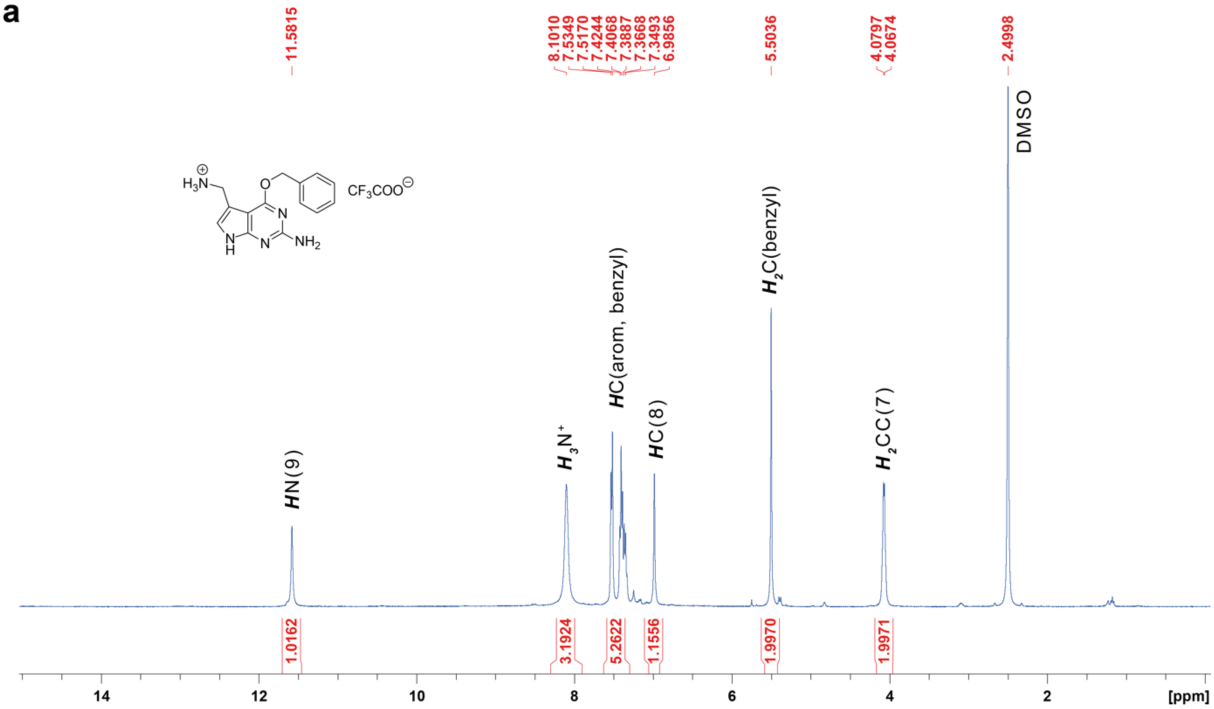

b

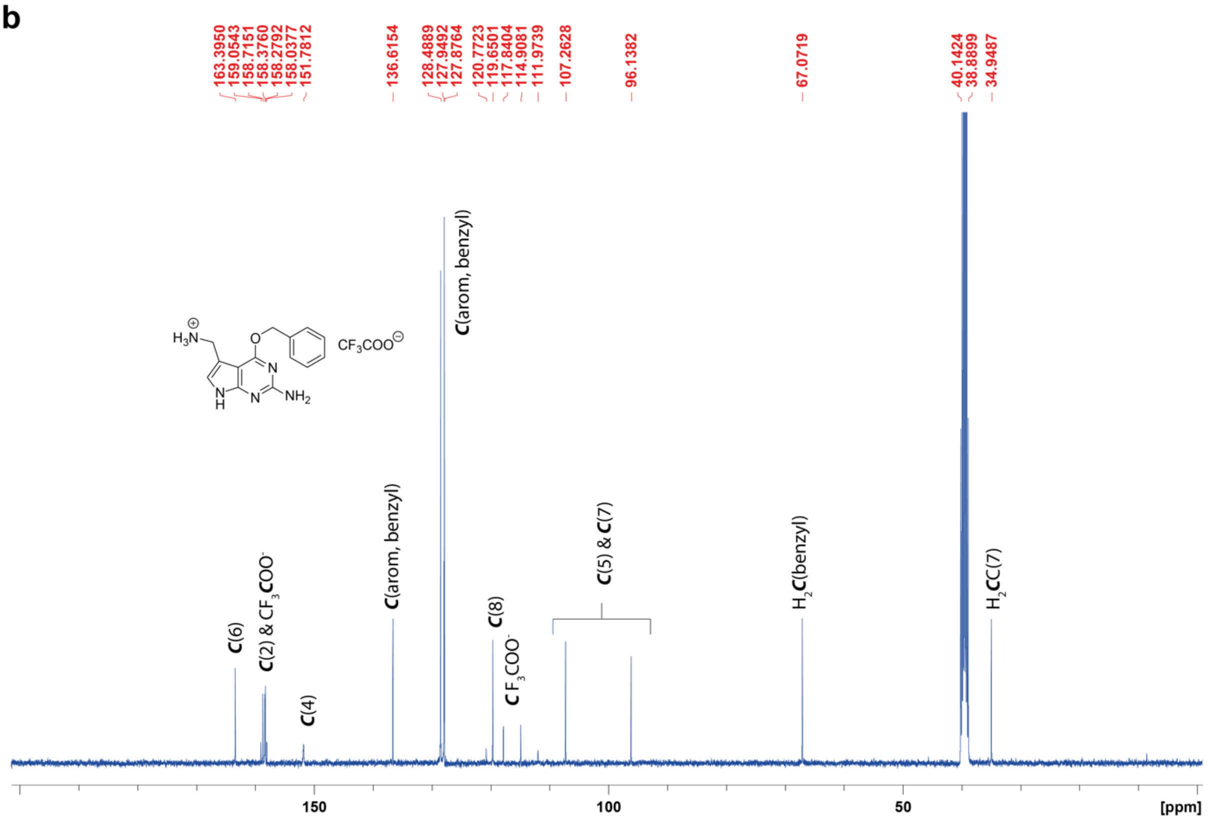

Supplementary Fig. 8. NMR spectroscopic analysis of bn[6]preQ[1] ligand. **a**, ^{1}H NMR spectrum. **b**, ^{13}C NMR spectrum.

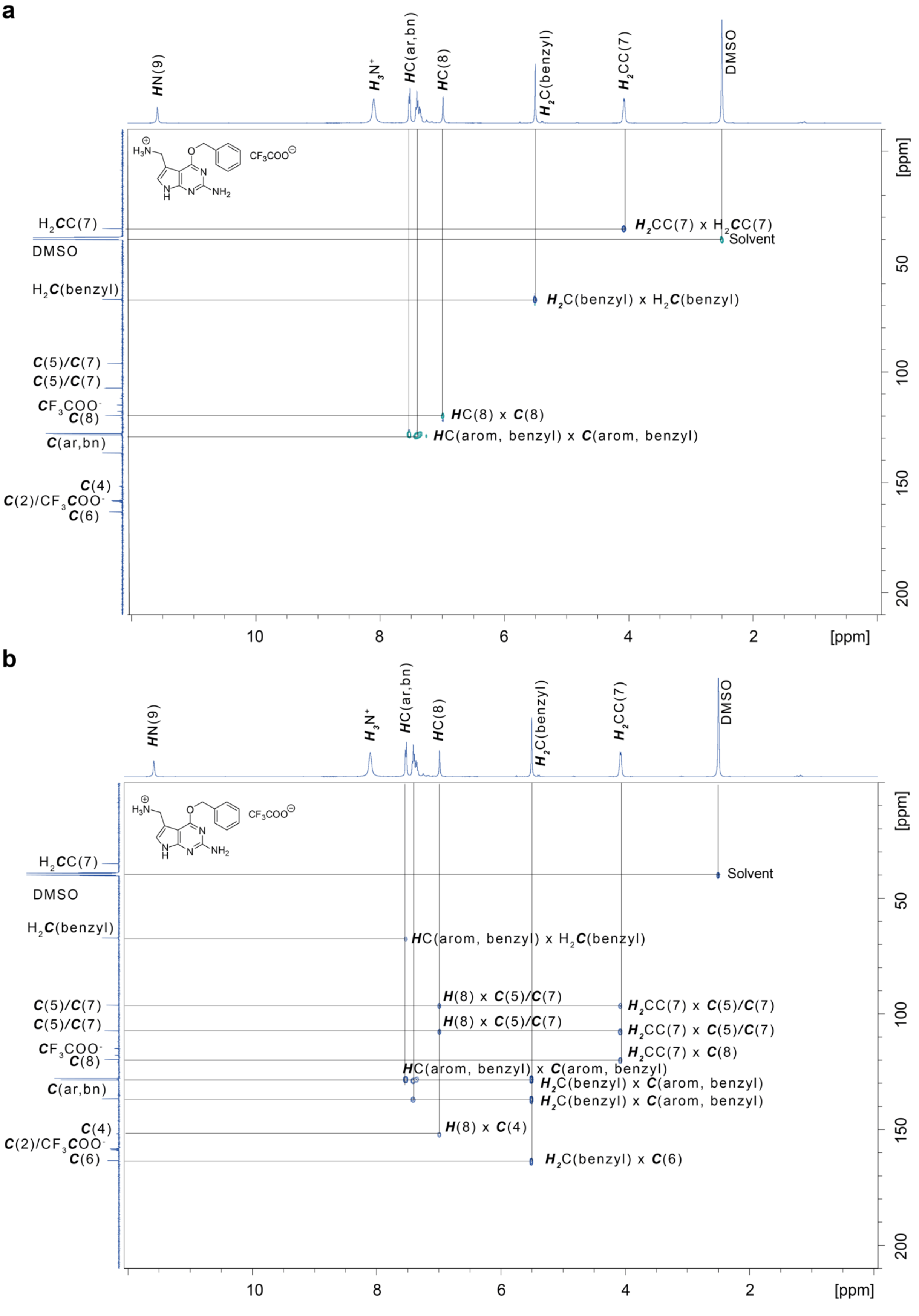

Supplementary Fig. 9. NMR spectroscopic analysis of bn^{6}preQ$_1$ ligand. **a**, ^{1}H,^{13}C HSQC NMR spectrum. **b**, ^{1}H,^{13}C HMBC NMR spectrum.

Supplementary Table 1. Synthetic RNAs and mass spectrometric analysis.

#	Name	Sequence (5' to 3')	nt	m.w. calculated	m.w. found
**1**	*Tt* preQ$_1$-I RNA wildtype	CUGGGUCGCAGUAACCCCAGUUAACAAAACAAG	33	10582.50	10582.49
**2**	*Tt* preQ$_1$-I RNA C15m^{3}C	CUGGGUCGCAGUAAm^{3}CCCCAGUUAACAAAACAAG	33	10596.50	10596.77
**3**	*Tt* preQ$_1$-I RNA C15m^{4}C	CUGGGUCGCAGUAAm^{4}CCCCAGUUAACAAAACAAG	33	10596.50	10596.71
**4**	*Tt* preQ$_1$-I RNA C15U	CUGGGUCGCAGUAAUCCCAGUUAACAAAACAAG	33	10583.49	10583.59
**5**	*Tt* preQ$_1$-I RNA G11dG	CUGGGUCGCAdGUAACCCCAGUUAACAAAACAAG	33	10566.50	10566.43
**6**	*Tt* preQ$_1$-I RNA G5C, G16C	CUGGCUCGCAGUAACGCCAGUUAACAAAACAAG	33	10582.50	10582.64
**7**	*Tt* preQ$_1$-I RNA ΔU12	CUGGGUCGCAGAACCCCAGUUAACAAAACAAG	32	10276.33	10276.66
**8**	*Tt* preQ$_1$-I RNA C15c^{3}C	CUGGGUCGCAGUAAc^{3}CCCCAGUUAACAAAACAAG	33	10581.50	10581.64
**9**	*B. subtilis* preQ$_1$-I RNA wildtype	AGAGGUUCUAGCUACACCCUCUAUAAAAAACUAA	34	10818.62	10818.53
**10**	*S. dysenteriae* preQ$_1$-I RNA wildtype	AUUGGGUUCCCUCACCCCAAUGGUUAAUCAAAAAGGU	37	11784.14	11784.81
**11**	*L. rhamnosus* preQ$_1$-II RNA wildtype	ACGACGAUACUUAUUUCCUUUGAUCGUCGUUAUUACU GGCAAAGCCACAAAGGAG	55	17563.59	17563.30
**12**	*Tt* preQ$_1$-I RNA U6C	CUGGGCCGCAGUAACCCCAGUUAACAAAACAAG	33	10581.51	10581.40
**13**	*Tt* preQ$_1$-I RNA A29G	CUGGGUCGCAGUAACCCCAGUUAACAAAGCAAG	33	10598.50	10598.63
**14**	2 strand construct short	UAACAAAACAAGG	13	4178.67	4178.60
**15**	2 strand construct long	CUGGGUCGCAGUAACCCCAGU	21	6687.08	6686.71
**16**	*Tt* preQ$_1$-I RNA C15m^{5}C	CUGGGUCGCAGUAAm^{5}CCCCAGUUAACAAAACAAG	33	10596.50	10596.85
**17**	*Tt* preQ$_1$-I RNA C15A	CUGGGUCGCAGUAAACCCAGUUAACAAAACAAG	33	10606.53	10606.36
**18**	*Tt* preQ$_1$-I RNA C15G	CUGGGUCGCAGUAAGCCCAGUUAACAAAACAAG	33	10622.53	10622.58
